# Cyclosporine Affects the Main Virulence Factors of *Cryptococcus neoformans* In Vitro

**DOI:** 10.3390/jof9040487

**Published:** 2023-04-18

**Authors:** Iara Bastos de Andrade, Dario Corrêa-Junior, Vinicius Alves, Maria Helena Galdino Figueiredo-Carvalho, Marcos Vinicius Santos, Marcos Abreu Almeida, Alessandro Fernandes Valdez, Leonardo Nimrichter, Rodrigo Almeida-Paes, Susana Frases

**Affiliations:** 1 Laboratório de Biofísica de Fungos, Instituto de Biofísica Carlos Chagas Filho, Universidade Federal do Rio de Janeiro, Rio de Janeiro 21941-902, Brazil; 2 Laboratório de Micologia, Instituto Nacional de Infectologia Evandro Chagas, Fundação Oswaldo Cruz, Rio de Janeiro 21941-902, Brazilmarcos.almeida@ini.fiocruz.br (M.A.A.);; 3 Instituto de Microbiologia Paulo de Góes, Universidade Federal do Rio de Janeiro, Rio de Janeiro 21941-902, Brazilnimrichter1@yahoo.com (L.N.); 4 Rede Micologia RJ, FAPERJ, Rio de Janeiro 21941-902, Brazil

**Keywords:** cyclosporine, *Cryptococcus neoformans*, capsule, calcineurin biology, virulence

## Abstract

This study aimed to investigate the effects of cyclosporine on the morphology, cell wall structure, and secretion characteristics of *Cryptococcus neoformans*. The minimum inhibitory concentration (MIC) of cyclosporine was found to be 2 µM (2.4 µg/mL) for the H99 strain. Yeast cells treated with cyclosporine at half the MIC showed altered morphology, including irregular shapes and elongated projections, without an effect on cell metabolism. Cyclosporine treatment resulted in an 18-fold increase in chitin and an 8-fold increase in lipid bodies, demonstrating changes in the fungal cell wall structure. Cyclosporine also reduced cell body and polysaccharide capsule diameters, with a significant reduction in urease secretion in *C. neoformans* cultures. Additionally, the study showed that cyclosporine increased the viscosity of secreted polysaccharides and reduced the electronegativity and conductance of cells. The findings suggest that cyclosporine has significant effects on *C. neoformans* morphology, cell wall structure, and secretion, which could have implications for the development of new antifungal agents.

## 1. Introduction

*Cryptococcus neoformans* is a pathogenic fungus that mainly causes infection in immunocompromised patients, such as people living with HIV/AIDS or transplant recipients [1]. Since the late 20th and early 21st centuries, there has been an increase in the immunocompromised population. These patients survive longer due to several recent advances in medicine, including with respect to surgical techniques, therapeutic regimens, and improved diagnoses [2]. Many patients undergo immunosuppressive therapy after organ transplantation and develop rheumatological disorders and autoimmune diseases [2]. Among the immunosuppressive drugs available, compounds that inhibit calcineurin, such as tacrolimus and cyclosporine, are commonly used in transplant recipients for immune tolerance of the implant [3]. They act by binding to cytoplasmic proteins belonging to the immunophilin family and thus interfering with the action of calcineurin. [4]. Calcineurin is a conserved Ca^2+^-calmodulin-activated protein phosphatase 2B belonging to the phosphoprotein phosphatase family of enzymes and is involved in the calcium-dependent signaling and regulation of several cellular processes of T cell development [5,6].

The calcineurin cascade may be an attractive target for drug development against eukaryotic pathogens [7,8]. In fungi, calcineurin has been shown to be required for the regulation of stress and growth responses, revealing its diverse and multifunctional roles in several species [6]. It is known that the calcineurin signaling pathway connects several features that are responsible for cryptococcal pathogenesis [9]. For example, calcineurin is required for hyphal elongation during mating and haploid fruiting in *C. neoformans* [10]. Earlier studies demonstrated that calcineurin is essential for the growth of *C. neoformans* at 37 °C, in 5% CO_2_, or at an alkaline pH, which are all conditions that mimic the host environment [11].

Studies evaluating the effects of calcineurin inhibition on the virulence factors of *C. neoformans* are still scarce. It is known that *C. neoformans* produces and secretes a variety of virulence factors that allow the fungus to infect and spread through a human host [12]. For example, polysaccharide capsules (impairment of host immune response), melanin production (protection against host temperature and oxidative stress), biofilms (protection against antifungal compounds and host immune response), and extracellular enzymes (degradation of tissue for nutrient acquisition) influence fungal adaptability and survival [13]. Therefore, we can conclude that effecting a decrease in this pathogen’s virulence factors is of paramount importance to attenuating its pathogenic power.

Based on the factors outlined above, the objective of this study is to evaluate whether cyclosporine affects the main virulence factors of *C. neoformans* in vitro. This will contribute to our understanding of the cell biology of this fungal pathogen and what happens to cryptococcal cells in humans with cryptococcosis that need to receive cyclosporine for the treatment of any other concomitant condition. It will also contribute to the body of knowledge regarding future anti-virulence strategies that can be studied to combat this important, basidiomycetous yeast.

## 2. Materials and Methods

Strain: The strain used throughout this work was the *C. neoformans* var. *grubii* H99 clinical isolate, which was kindly provided by Professor Arturo Casadevall of Johns Hopkins Bloomberg School of Public Health, Baltimore, MD, United States. This is a wild-type strain available in the American Type Culture Collection (ATCC catalog number 208821).

Antifungal activity: Experiments for determination of minimal inhibitory concentrations (MICs) were performed using the broth microdilution method following the recommendations of the Brazilian Committee on Antimicrobial Susceptibility Testing (BrCAST) [14]. Cyclosporine (Sigma-Aldrich Co., Ltd., San Luis, MO, USA) was serially diluted (20 to 0.03 µM/24 to 0.036 µg/mL) [11,15] in RPMI 1640 (pH 7.0, with 2% glucose) buffered with 3-(N-Morpholino) propane sulfonic acid in 96-well plates. The inoculum of the fungal isolate was prepared as previously described [14]. The plate was incubated at 37 °C for 48 h. MICs were determined as the lowest cyclosporine concentration capable of completely inhibiting fungal growth (which was verified by the absence of visible turbidity in the wells). Controls consisting of cryptococcal cells cultured without cyclosporine and culture media without fungal cells and cyclosporine were included in the experiments. All experiments described below were performed with cells treated with half cyclosporine MIC to observe the effect of the drug on living cells.

Characterization of cell viability: Cyclosporine toxicity for the *C. neoformans* H99 strain was evaluated through a cell viability assay performed using the CyQUANT™ XTT kit (Thermo Fisher, Hillsboro, OR, USA), which was used according to the manufacturer’s instructions. This colorimetric assay investigates the reduction of the hydroxide metabolite of 2,3-bis(2-methoxy-4-nitro-5-sulfophenyl)-5[(phenyl-amino)carbonyl]-2H-tetrazolium (XTT) to a soluble brown product in water, namely, formazan. Aerobically breathing (viable) cells convert the XTT compound into an orange-colored product (formazane) [16].

Morphologic evaluation: To analyze the micromorphology of cyclosporine-treated and untreated cells, 10 µL samples of the cultures were harvested after 7 days of incubation at 37 °C and placed on a microscope slide with 10 µL of Indian ink and observed using an Axiolab optical microscope (Zeiss) with 400× magnification [15].

Chitin quantification: Treated and untreated cells were labeled with 10 µg/mL of Uvitex2B (Polysciences Inc., Warrington, PA, USA) incubated at 37 °C for 30 min. Yeasts suspended in phosphate-buffered saline (PBS) without Uvitex2B were used as an autofluorescence control. Then, cells were washed three times with PBS to remove excess dye. For fluorescence quantification, cells were adjusted to a concentration of 1 × 10^4^ cells/mL and read according to their cytometer flow rates (BD LSRFortessaTM X-20, BD Biosciences, San Jose, CA, USA) at a 350 nm excitation wavelength and a 435 nm emission wavelength. The untreated population was mapped without marking and its size was delimited. This gate was used for cells labeled with and without treatment with cyclosporine. The mapping of the population (n = 10,000 events) was conducted to determine the size and log of blue fluorescence using a single-parameter histogram. Results were expressed as mean fluorescence intensity (MFI) divided by cell size [17]. Additionally, cells were observed under an Axio Observer fluorescence microscope (Zeiss, Jena, Germany) for morphological evaluation [18,19].

Lipid bodies estimation: Fungal cells treated and not treated with cyclosporine were fixed in paraformaldehyde (10^6^ cells), labeled with Nile red (Sigma-Aldrich, St. Louis, MO, USA), at 5 μg/mL for 30 min at room temperature. This fluorochrome has an affinity for neutral lipids present in lipid aggregates. Yeasts suspended in PBS were included as an autofluorescence control. Subsequently, cells were washed three times in PBS, adjusted to a concentration of 1 × 10^4^ cells/mL, and read using a flow cytometer (BD LSRFortessaTM X-20, BD Biosciences, San Jose, CA, USA) operating at an excitation wavelength of 552 nm and a 636 nm emission wavelength. The untreated population was mapped without marking and its size was delimited. This gate was used for cells labeled with and without treatment with cyclosporine. The mapped population (n = 10,000 events) was analyzed to determine the size and log of red fluorescence using a single-parameter histogram. Results were expressed as the mean of the fluorescence intensity (MFI) divided by the cell size [19].

Capsule Size: Cells were grown in a minimal capsule-inducing medium (MM) (15 mM glucose, 10 mM MgSO_4_, 29.4 mM KH_2_PO_4_, 13 mM glycine, 3 μM thiamine, and at pH 5.5) that was either supplemented or not with cyclosporine for 7 days at 35 °C. To measure capsule thickness, cells were centrifuged at 6708× *g* for 10 min, negatively stained with India ink, and then imaged in an AXIO Lab.A1 light microscope (ZEISS, Jena, Germany). The capsule thickness (i.e., the distance between the cell wall and the outer limit of the capsule) was measured in a minimum group of 100 cells using the ImageJ software 1.8.0 g (http://rsb.info.nih.gov/ij/ (accessed on 18 April 2023), National Institutes of Health (NIH), Bethesda, MD, USA) [20].

Quantification of secreted polysaccharide: To quantify the secreted cryptococcal polysaccharide, cells were grown in the same minimal capsule-inducing medium described above, which was either supplemented or not with cyclosporine for 7 days at 35 °C. Based on their ability to self-aggregate, secreted polysaccharides accumulate in the supernatant of cultures that have been isolated by centrifugation (6708× *g* for 10 min), as described by Nimrichter et al. (2007) [21]. The final solution was quantified by the phenol-sulfuric colorimetric method, for which glucose was used as a standard [22].

Extraction and concentration of secreted polysaccharides (PS): For the extraction of secreted polysaccharides (PSs), cells were grown in 15 mL of liquid Sabouraud dextrose medium (Kasvi, Espanha) on a rotary shaker for 24 h at 37 °C. After that, 1 mL of this culture was centrifuged and resuspended in 500 mL of the minimal capsule-inducing medium described above in the absence or presence of cyclosporine at a concentration of 1 µM. After seven days under constant agitation at 37 °C, the cultures were centrifuged at 1677× *g* for 15 min in order to separate cells and the supernatant. The obtained supernatant, which contains all the secreted polysaccharide, was concentrated through ultrafiltration in the AMICON system (Millipore, Danvers, MA, USA) using a membrane that retains any structure with a molecular mass equal to or greater than 10 kDa. After this process, the PSs were stored at 4 °C for later analysis. Subsequently, the PSs were weighed to reach a final concentration of 10 mg/mL. Then, the samples were analyzed using Zeta Potential and conductance techniques, dynamic light scattering, and passive microrheology using the NanoBrook Omni (Brookhaven Instruments Corp., Holtsville, NY, USA).

Zeta Potential (ξ) and conductance measurements: Cells and PSs treated with or without cyclosporine were obtained as described above. Measurements were taken on a NanoBrook Omni Zeta potential analyzer (Brookhaven Instruments Corp., Holtsville, NY, USA), as previously described by Frases et al. (2009) [23]. Ten measurements for each sample were taken at 25 °C.

Dynamic Light Scattering (DLS): A solution containing 10 mg/mL of PS in deionized water secreted by cells treated or untreated with cyclosporine was analyzed in order to measure the effective diameter of the polysaccharide fibers as previously described by Frases et al. (2009) [23]. Measurements were taken using NanoBrook Omni (Brookhaven Instruments Corp., Holtsville, NY, USA).

Passive microrheology: Viscoelastic properties of a 10 mg/mL solution of PS in deionized water secreted by cells treated and not treated with cyclosporine were analyzed. Viscous modulus (G″), elastic modulus (G′), and complex viscosity (η*) values were obtained with a 1 µm polystyrene microsphere probe (Polysciences, Inc., Warrington, USA) as described previously by Araujo et al. (2019) [24]. Deionized water was employed as an experimental control, whose physicochemical properties have already been well elucidated. Measurements were performed on the NanoBrook Omni (Brookhaven Instruments Corp., Holtsville, NY, USA).

Determination of phospholipase activity: Extracellular phospholipase activity was determined using egg yolk agar plates. The test medium consisted of Sabouraud 4% dextrose agar (SDA, Kasvi, Espanha) containing 1 M of NaCl, 5 mM CaCl_2_, and 8% sterile egg yolk emulsion (pH 7.0), as previously described by Price et al. (1982) [25]. To determine phospholipase activity, first, the fungal cells were cultured in the presence and absence of a subinhibitory cyclosporine concentration in MM for 7 days. Subsequently, fungal cells cultured with 48 h in SDA and aliquots (10 µL) of 10^5^ cells/mL were placed on the surface of the egg yolk agar medium and incubated at 37 °C for 7 days. Colony diameter (a) and colony diameter plus hydrolysis/precipitation zone (b) were measured, and production was expressed as Pz value (a/b) (as previously described) [25]. The Pz value was scored according to four categories: Pz of 1.0 indicated no production; Pz between 0.999 and 0.700 indicated weak producers; Pz between 0.699 and 0.400 corresponded to good producers; and Pz less than 0.399 indicated excellent producers [25].

Urease Production: To verify urease production, cells were cultured in MM for 7 days in the presence and absence of cyclosporine at subinhibitory concentrations. Then, both fungal cells (treated and untreated) were grown in Christensen urea broth [26] in the presence and absence of cyclosporine at the same concentration. Equivalent suspensions of yeast cells from each condition were adjusted to a concentration of 10^6^ cells/mL and used for inoculation. A volume of 500 μL of the suspension was inoculated into 4.5 mL of Christensen urea broth and the tubes were then incubated with agitation (150 rpm) at 37 °C. At the end of the five days of incubation, the tubes were centrifuged, and a volume of 100 μL of the supernatant was transferred to a 96-well polystyrene flat bottom plate (Corning, Tewksbury, MA, USA); this procedure was performed in triplicate. *Candida albicans* was used as a negative control. The absorbances of the samples were read on a SpectraMax plus 384 spectrophotometer (Molecular Devices, San José, CA, USA) at 559 nm, as previously described by Almeida-Paes et al. (2015) [27]

Data analyses: GraphPad Prism 9.0 software was used for statistical analysis with appropriate parametric or non-parametric tests after verification of data normality using the Shapiro–Wilk test. A *p*-value < 0.05 was considered significant for all statistical analyses.

## 3. Results

**Figure 1:**
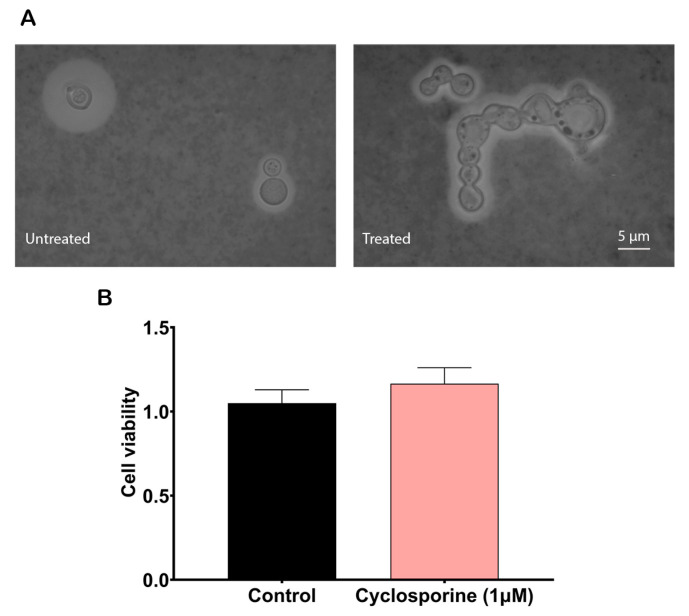
Effects of cyclosporine on cryptococcal cells. (**A**) Morphological alterations of *C. neoformans* induced by cyclosporine. Yeasts were stained with Indian ink to show their polysaccharide capsules. Scale bar: 5 µm. (**B**) Effect of cyclosporine on the metabolic activity of *C. neoformans*, measured using the XTT test. Statistical significance was determined via a *t*-test, which did not find statistical differences (*p*-value = 0.1778).

To analyze cyclosporine’s effects on the *C. neoformans* cells, we first calculated its MIC for the H99 strain, which was 2 µM (2.4 µg/mL). When the yeasts were treated at half cyclosporine MIC, we observed that this drug could alter the morphology of *C. neoformans*. Some of the yeast cells lost their regular spherical morphologies and presented an irregular shape with continuous buds that did not break away from the mother cell, yielding clusters of cells with elongated projections (Figure 1A). In addition, to evaluate whether cyclosporine at this concentration is toxic to *C. neoformans*, the XTT test was performed. The results showed that the drug did not alter cell metabolism in a statistically significant way at a concentration of 1 µM (1.2 µg/mL) (Figure 1B), proving that under this concentration, *C. neoformans* yeasts exposed to cyclosporine are viable and metabolically active, thus supporting the execution of the following experiments.

**Figure 2:**
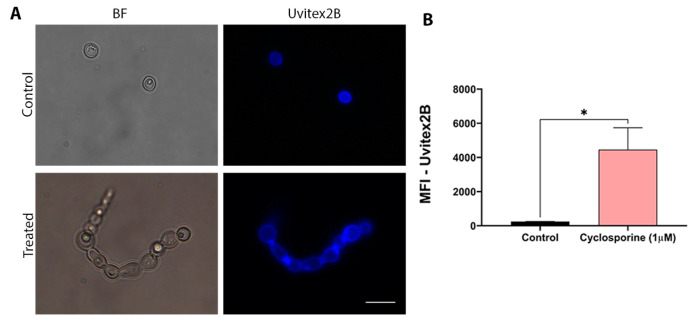
Effects of cyclosporine on chitin production by *Cryptococcus neoformans*. (**A**) Fluorescence electron microscopy of untreated and treated cells with 1 µM cyclosporine. BF: bright field; Uvitex2B: dye used for marking chitin. Scale bar: 10 µm. (**B**) Analysis of the mean fluorescence intensity (MFI) of untreated *C. neoformans* cells and those treated with 1 µM cyclosporine and labeled with Uvitex2B, a chitin cell wall marker. Statistical significance was determined via a *t*-test (* *p*-value = 0.04).

Chitin is one of the main components of the fungal cell wall. Thus, changes in chitin concentrations may indicate structural changes in the cell wall. Therefore, chitin labeling was carried out to assess whether cyclosporine can alter the cell wall structure of *C. neoformans*. Cells were treated with cyclosporine and labeled with Uvitex2B. Unlabeled cells did not present autofluorescence. To evaluate the structural modifications that cyclosporine can induce in the cell wall of *C. neoformans*, fluorescence microscopy was performed, for which the cells were stained with Uvitex2B. (Figure 2A). Quantification of fluorescence, which was determined using flow cytometry, demonstrated an 18-fold increase in the amount of chitin in the cyclosporine-treated cells (Figure 2B, *p*-value = 0.0433).

**Figure 3:**
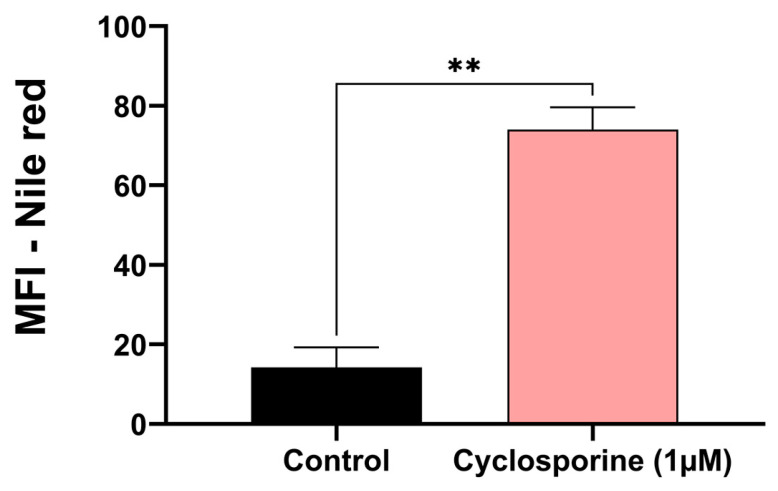
Analysis of the mean fluorescence intensity (MFI) of untreated *C. neoformans* cells and those treated with 1 µM of cyclosporine and stained with Nile red, a lipid body marker. Statistical significance was determined via a *t*-test (** *p*-value = 0.007).

Eukaryotic cells can form lipid bodies, which are composed of neutral lipids. They act as a reservoir of energy and participate in the formation and maintenance of membranes [19]. For this reason, we investigated the presence of lipid bodies in *C. neoformans* treated with cyclosporine. Our results demonstrated that there was an eight-fold increase in the number of lipid bodies in the cyclosporine-treated cells (Figure 3, *p*-value = 0.007).

**Figure 4:**
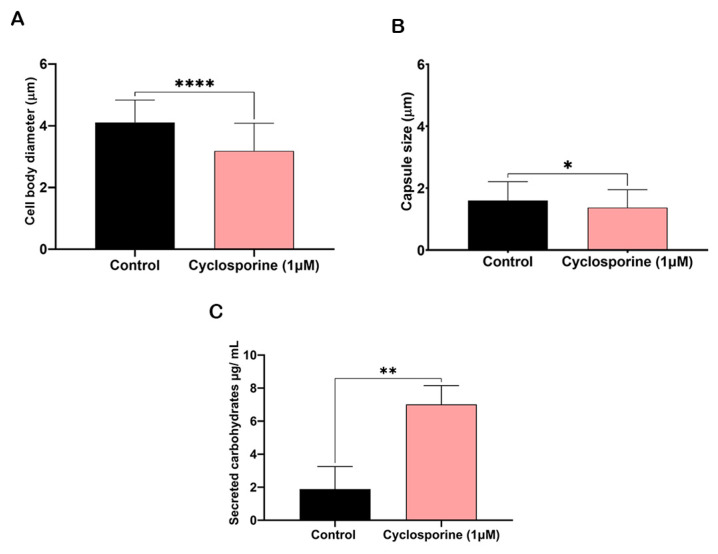
Measurements of cellular bodies (**A**), capsules (**B**), and secreted carbohydrates (**C**) of *C. neoformans* cells treated and untreated with 1 µM of cyclosporine. **** *p*-value < 0.001, * *p*-value = 0.02, ** *p*-value = 0.0075. Student’s *t* test was used for statistical analysis.

The cells treated with cyclosporine presented an irregular appearance; therefore, only isolated cells without aberrant protuberances were measured. Cryptococcal cells treated with cyclosporine presented a reduction in both cell body and polysaccharide capsule diameters (Figure 4A and 4B, respectively). Cyclosporine reduced the cells’ body diameters by 22.47%, resulting in a statistically significant difference between the treated and treated cells (*p*-value < 0.0001), and reduced the diameter of the polysaccharide capsules by 14.25%, resulting in a another statistically significant difference between the treated and untreated cells (*p*-value = 0.02). We suspected that the polysaccharide that is not incorporated into the cryptococcal capsule may be secreted into the extracellular medium. Then, to analyze whether the drug can alter carbohydrate secretion, we first quantified the carbohydrates in the supernatant of the cultures of the cells treated or untreated with cyclosporine. The drug interfered with the number of total carbohydrates secreted (Figure 4C), with 3.71 times more carbohydrates being secreted when the cells were treated with cyclosporine.

**Figure 5:**
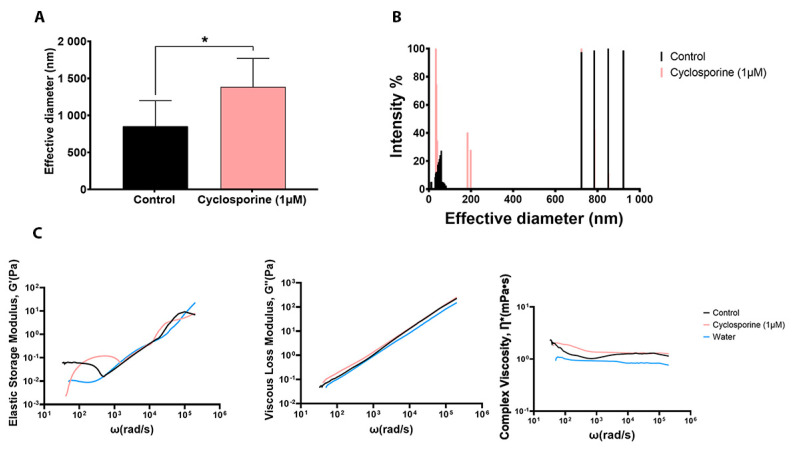
Calculation of the effective diameter (**A**) and size distribution (**B**) of PS secreted by *C. neoformans* in cells treated and not treated with 1 µM of cyclosporine. * *p*-value = 0.0499. Statistical significance was determined using Student’s *t* test. (**C**) Passive microrheology of secreted polysaccharides (PS) from *C. neoformans* in the presence and absence of cyclosporine. Elastic modulus G′(Pa), left; viscous modulus G”(Pa), middle; and viscous complex Ƞ*(mPa•s), right, were measured.

The analysis of the secreted PS diameter revealed that the PSs produced by the cells treated with cyclosporine presented a larger diameter (*p*-value = 0.0499) (Figure 5A) and a different PS fiber distribution when compared to the PSs of the untreated cells (Figure 5B). Analysis of the mechanical spectra (elastic storage modulus, G′, and viscous loss modulus, G″) of the PSs produced by *C. neoformans* showed that both G′ and G″ showed little variation with respect to frequency. Complex viscosity curves showed that the complex viscosity of the PSs from the cells treated with cyclosporin was higher than that of the untreated cells and water. In addition, the viscosity of the complex remained constant with different frequencies under different conditions. Thus, we can conclude that cyclosporine was able to increase the viscosity of the secreted PSs in relation to the medium (Figure 5C).

**Figure 6:**
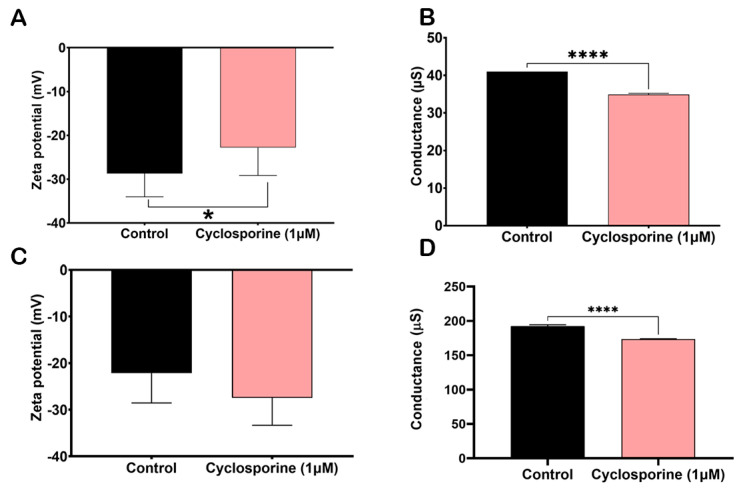
Effect of cyclosporine on zeta potential (**A**) and conductance (**B**) of *C. neoformans* cells. * *p*-value = 0.03 and **** *p*-value < 0.001; statistical significance determined by *t* test. Effect of cyclosporine on the zeta potential (**C**) and conductance (**D**) of PSs from *C. neoformans*. **** *p*-value < 0.001; statistical significance determined using Student’s *t* test.

The Zeta potential is a tool that allows for the measurement of the electronegativity changes induced by cyclosporine on the cell surfaces and PS of *C. neoformans*. The cells treated with cyclosporine were less electronegative when compared to the control cells (Figure 6A, *p*-value = 0.0371). Furthermore, conductance, a tool that allows for the measurement of ionic charges, was lower in the cells treated with the drug, presenting significant differences (Figure 6B, *p*-value< 0.0001). However, the electronegativity of the PSs treated with cyclosporine did not differ significantly when compared to the control PSs (Figure 6C, *p*-value = 0.07). Furthermore, PS conductance was also lower when treated with the drug, with significant differences (Figure 6D, *p* value < 0.0001).

**Figure 7:**
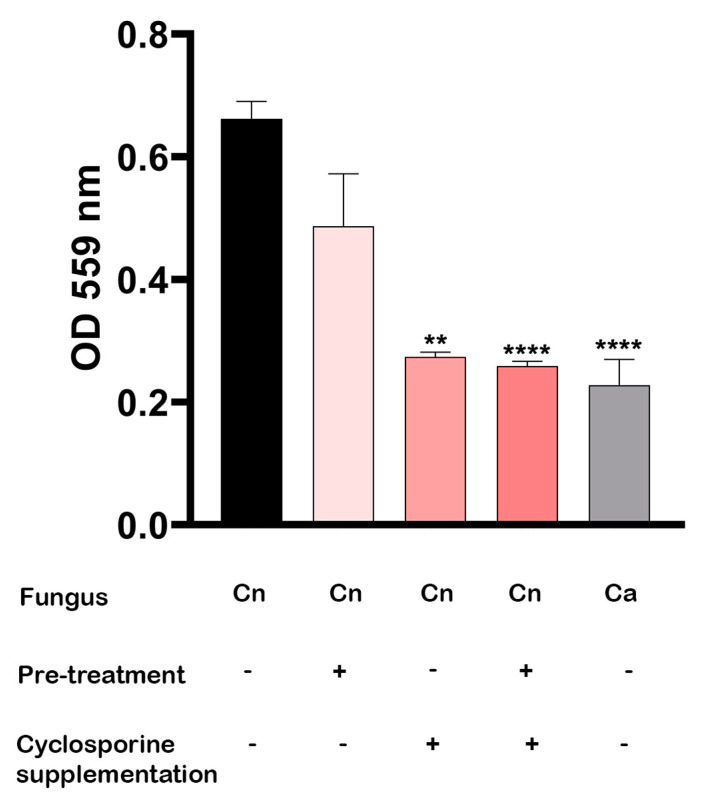
Urease production, measured according to optical density (OD) at 559 nm, of *C. neoformans* previously treated or not with 1 µM cyclosporine in Christensen’s urea broth. *C. albicans* was used as negative control. ** *p*-value = 0.0038 and **** *p*-value < 0.00001; statistical significance was determined using Kruskal–Wallis test.

When the optical densities of the culture supernatants at 559 nm were determined, we observed that the presence of cyclosporine in urea broth is the most efficient way to inhibit urease secretion (Figure 7). After five days of incubation at 37 °C, the *C. neoformans* cultures supplemented with cyclosporine showed a significant reduction in urease secretion in both the previously treated cells (*p*-value < 0.0001) and untreated cells (*p*-value = 0.0038). The cells that had been previously treated with cyclosporine and that were added to the drug-free culture medium also showed a reduction of 26.43% in urease production, but without presenting a statistically significant difference (*p*-value = 0.5838).

**Figure 8:**
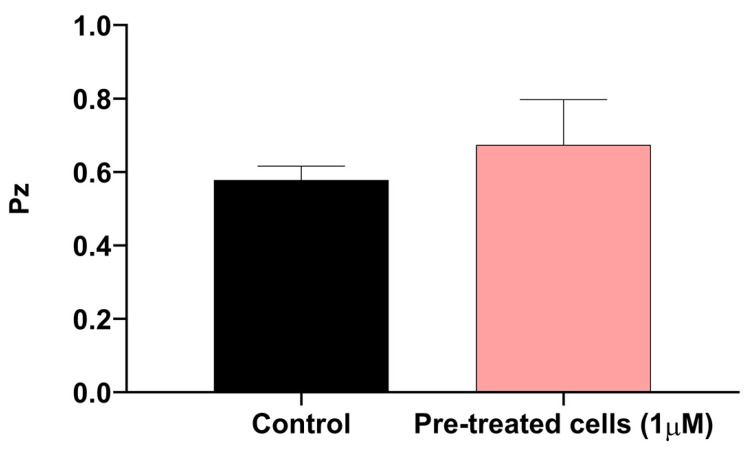
Phospholipase secretion by *C. neoformans* treated and not treated with 1 µM of cyclosporine. Statistical significance determined by *t* test without significant differences.

We decided to investigate the production of phospholipases in the cyclosporine-treated and untreated *C. neoformans* cells through an agar plate assay (Figure 8). Under our experimental conditions, the mean Pz of the control cells was 0.57, while that of the treated cells was 0.67, indicating that both could be considered good phospholipase producers. Despite the decrease in enzymatic activity, we did not observe significant differences between the treated and untreated cells (*p*-value = 0.1891) (Figure 8).

## 4. Discussion

Calcineurin is a serine–threonine-specific, Ca^2+^–calmodulin-activated protein phosphatase that is important in the mediation of cell-stress responses [28]. It is a heterodimer that is composed of a catalytic A and regulatory B subunit, and its activation requires association between the two subunits. Following the mobilization of Ca^2+^ stores, the catalytic subunit is bound by Ca^2+^–calmodulin, thus freeing the active site from occlusion by a now-displaced autoinhibitory domain [29]. In T cells, calcineurin dephosphorylates the cytoplasmic component of the transcription factor nuclear factor of activated T-cells (NF-AT), which is necessary for interleukin (IL)-2 transcription and T-cell activation [9]. Calcineurin is the target of the immunosuppressants cyclosporine and tacrolimus [30]. The interactions between cyclosporine or tacrolimus and calcineurin inhibit NF-AT nuclear translocation, thereby preventing NF-AT from binding to its target genes [31].

The biology of fungal calcineurin has gained importance over the years. It has been reported to be necessary for fungal growth and virulence [7]. In animal models, the defective growth of a *C. neoformans* calcineurin mutant at high temperatures showed that calcineurin is critical for pathogenicity. Furthermore, mutant growth is profoundly inhibited by physiological CO_2_ concentrations and alkaline conditions. Therefore, the calcineurin pathway in *C. neoformans* may coordinate the response to general environmental stresses to allow growth in mammalian hosts [9].

Previous in vitro studies have demonstrated the anticryptococcal activity of cyclosporine [11,32,33]. There is no consensus in the literature as to whether cyclosporine protects patients from cryptococcosis. In a study employing an experimental rabbit infection model, the mean time to death was approximately two weeks with cyclosporine, while it was approximately four weeks with cortisone [34]. The cited authors concluded that for some mycotic infections, there may be a precarious balance between the effects of immunosuppression and the direct antifungal activity of cyclosporine. In this work, it was suggested that humans receiving cyclosporine are likely to experience an increased incidence of cryptococcal infection [34].

On the other hand, another study showed that the survival of mice was prolonged in animals that received a prophylactic treatment with immunosuppressive doses of cyclosporine after *C. neoformans* inoculations. Furthermore, mice treated with cyclosporine cleared *C. neoformans* from their lungs more quickly than the control mice [32]. In another study by the same group, the authors determined whether an established infection by *C. neoformans* could be treated with cyclosporine. The survival of the infected mice that received cyclosporine was prolonged in both immunologically intact and congenitally T-cell-deficient mice [33].

Our results confirmed this in vitro antifungal activity, employing a similar MIC to that found in other studies [11]. Specifically, for *C. neoformans*, the mechanism currently described for the antifungal activity of calcineurin inhibitors is the ability to affect growth at high temperatures in addition to inhibiting hyphal elongation during mating and haploid fruiting [11].

Our group recently described that cyclosporine could alter the morphology of *C. neoformans* [15]. The present study demonstrated that the observed level of capsule reduction is due to the inability to assemble polysaccharides in the cryptococcal capsule, which are instead secreted in the culture supernatant. This effect may be related to the attenuation of the virulence of this pathogen, considering that the polysaccharide capsule is one of the main virulence factors of *C. neoformans* [35,36,37]. Differences in zeta potential suggest that the decrease in the electronegativity of the cyclosporine-treated cells may be due to a decrease in exposure to the acidic glucuronide present in the polysaccharide capsules. These differences may be related to a type of modulation that cyclosporine induces in the composition of the polysaccharide capsule since the capsular size is also altered. We believe that this result reinforces the hypothesis of the attenuation of the virulence of *C. neoformans* via the effect of cyclosporine [38]. Furthermore, the degree of conductance was lower in the cells treated with the drug, indicating that cyclosporine can decrease the anionic charges generated by the glucuronic acid present in the polysaccharide capsule.

Chitin is required for *C. neoformans* [39] and is a major component of the fungal cell wall [19]. The deacetylation of chitin to chitosan causes a significant change in the physicochemical properties of the former polymer [40]. Chitosan is a critical component of the cryptococcal cell wall during infections in mammals [39]. In this work, we observed that there were changes in the amount of chitin in the cells treated with cyclosporine. These results may indicate that the drug can induce structural changes in the cell wall, as seen in fluorescence microscopy. We infer that the increase in chitin labeling is due to a possible stress response induced by the drug. In addition, it was seen that there was also an increase in the quantity of lipid bodies. Eukaryotic cells can form lipid bodies, which are composed of neutral lipids. Neutral lipids act as an energy reservoir and participate in the formation and maintenance of the membrane [19]. The increased formation of lipid bodies, as seen in our results, may indicate a response to some type of stress.

Urease is also an important virulence factor in *C. neoformans* that allows for the migration of the fungus to the brain parenchyma, a process that may be impeded by urease inhibitors [41]. Our results showed that cyclosporine is capable of inhibiting urease production, which correlates calcineurin’s biological characteristics with nitrogen metabolism in *C. neoformans* and with a possible impact on the pathogenesis of cryptococcal meningitis. Another main hydrolytic enzyme secreted by *C. neoformans* is phospholipase, which is implicated in the initiation and spread of infection [42]. Our results demonstrate that cells previously treated with cyclosporine showed limited inhibition of the secretion of this enzyme, even if it was not statistically significant.

Our results suggest that cyclosporine is able to attenuate several virulence factors in *C. neoformans*, such as capsule polysaccharide production and urease and phospholipase secretion. However, the limitation of this study is that we used only one reference strain. For this reason, we believe that it is necessary to study the effects of cyclosporine on other strains of *C. neoformans*. The study of the effects of immunosuppressants on fungal properties can aid the management of patients with fungal infections who need to use these drugs to treat other pathologies. In vivo experiments are required to understand the cryptococcal pathogenesis of patients using cyclosporine.

We conclude that cyclosporine is able to reduce the virulence of *C. neoformans*; however, it also reduces the host’s immune response. Decreased immunity appears to be more important in the pathogenesis of cryptococcosis during the use of cyclosporine [34]. Therefore, it is imprudent to repurpose cyclosporine in its current formulations for cryptococcosis treatment. However, cyclosporin-derived molecules that retain the ability to decrease the virulence of *C. neoformans* and do not affect host immunity may be developed in future research to help to treat this life-threatening fungal infection.

## Data Availability

The data presented in this study are available within the article.
